# Two Different Protocols for Knee Joint Motion Analyses in the Stance Phase of Gait: Correlation of the Rigid Marker Set and the Point Cluster Technique

**DOI:** 10.1155/2012/586348

**Published:** 2012-09-13

**Authors:** Takashi Fukaya, Hirotaka Mutsuzaki, Hirofumi Ida, Yasuyoshi Wadano

**Affiliations:** ^1^Department of Physical Therapy, Faculty of Health Sciences, Tsukuba International University, 6-8-33 Manabe, Ibaraki, Tsuchiura 300-0051, Japan; ^2^Department of Orthopedic Surgery, Ibaraki Prefectural University of Health Sciences, 4669-2 Ami, Ibaraki, Ami-machi 300-0394, Japan; ^3^Department of Human System Science, Tokyo Institute of Technology, 2-12-1 Oh-okayama, Meguro, Tokyo 152-8550, Japan

## Abstract

*Objective*. There are no reports comparing the protocols provided by rigid marker set (RMS) and point cluster technique (PCT), which are similar in terms of estimating anatomical landmarks based on markers attached to a segment. The purpose of this study was to clarify the correlation of the two different protocols, which are protocols for knee motion in gait, and identify whether measurement errors arose at particular periods during the stance phase. *Methods*. The study subjects were 10 healthy adults. All estimated anatomical landmarks were which their positions, calculated by each protocol of the PCT and RMS, were compared using Pearson's product correlation coefficients. To examine the reliability of the angle changes of the knee joint measured by RMS and the PCT, the coefficient of multiple correlations (CMCs) was used. *Results*. Although the estimates of the anatomical landmarks showed high correlations of >0.90 (*P* < 0.01) for the *Y*- and *Z*-coordinates, the correlations were low for the *X*-coordinates at all anatomical landmarks. The CMC was 0.94 for flexion/extension, 0.74 for abduction/adduction, and 0.71 for external/internal rotation. *Conclusion*. Flexion/extension and abduction/adduction of the knee by two different protocols had comparatively little error and good reliability after 30% of the stance phase.

## 1. Introduction

Gait analysis with motion analysis based on a camera system has been widely applied both clinically and in research. It is used to assess changes over time in patients and to evaluate differences in their gait patterns compared with those of normal subjects. Gait analysis for patients with problems such as osteoarthritis and ligament injury of the knee has been previously reported [1–3]. However, measurement errors are caused by the method used to attach the reflective markers to the body in gait analysis with motion analysis, and these errors influence the reliability of the results. In previous studies, sufficiently reliable results were not obtained for movement of either the frontal or horizontal plane, although the reliability was comparatively high for movement of the sagittal plane of the knee joint [4, 5].

A set of at least three noncollinear reflective markers on each segment is required to define a rigid body in three-dimensional space. While measurement of marker sets mounted on bone pins [6] is comparatively accurate, the procedure is invasive and difficult to use. Although the reflective markers are attached directly to the skin in skin-mounted marker sets [7], soft tissue artifacts are increased by muscle contraction and the impact of the initial contact during the stance phase, leading to the development of measurement errors when the joint angle and joint moment are calculated.

The rigid marker set (RMS), a method to estimate the positions of anatomical landmarks in motion by calibrating anatomical landmarks from the three markers mounted on rigid plates in the standing position, has been previously reported [8–10]. The method using markers mounted on rigid plates is possible to prevent the independent movement of each marker compared with the method using skin-mounted marker sets.

The point cluster technique (PCT) reported by Andriacchi et al. [11], which is a calculation method that reduces the measurement error caused by artifacts of each skin marker, was used clinically as a noninvasive method [12, 13]. The PCT involves attachment of multiple reflective markers (usually about 5–20) on the thigh and shank together with anatomical landmarks. In this technique, the three-dimensional movement of the knee joint is calculated from the estimated positions of the femur and tibia bones in vivo, where a principal axis transformation for the segment marker clusters is used to define the local reference system for this estimation. Andriacchi et al. [11] computationally simulated that the PCT could reduce the influence of skin movement artifacts. They also demonstrated that the obtained data were comparable to the relative movement of the thigh and shank bones during walking reported in a previous study [6]. However, it is also considered that the PCT is insufficient to catch the three-dimensional motion of the knee joint during measurements [14]. Both RMS and the PCT were devised as protocols to reduce measurement errors caused by skin movement artifacts. Although these protocols are similar in terms of estimating anatomical landmarks based on markers attached to a segment to describe the three-dimensional motion of the knee joint, there are no previous reports of studies comparing the protocols provided by RMS and the PCT. Evaluation of the results obtained by these two protocols and examination of the estimates of the anatomical landmarks will reveal the influences on the results of both protocols and identify the periods of the stance phase in which errors occur.

The purpose of this study was to clarify the correlation of the two different protocols, RMS and the PCT. For this purpose, we examined changes in the estimates of anatomical landmarks and knee joint angles obtained by RMS and the PCT as protocols for knee joint motion in gait analyses and identified whether measurement errors arose at particular periods during the stance phase.

## 2. Methods

### 2.1. Subjects

The study subjects were 10 healthy adults (7 males and 3 females; mean age ± SD, 29.2 ± 5.0 years; mean height ± SD, 1.70 ± 0.12 m; mean mass ± SD, 67.4 ± 9.5 kg; mean BMI ± SD, 23.3 ± 2.4 kg/m^2^) who had neither orthopedic disease of the lower limbs, including ligament injury or bone/spinal fracture, nor neurological impairment and had no limitations in their activities of daily life. All subjects provided written informed consent prior to any assessment. Ethical approval for this study was obtained from the Ibaraki Prefectural University of Health Sciences Ethics Committee.

### 2.2. Procedure

**Figure 1:**
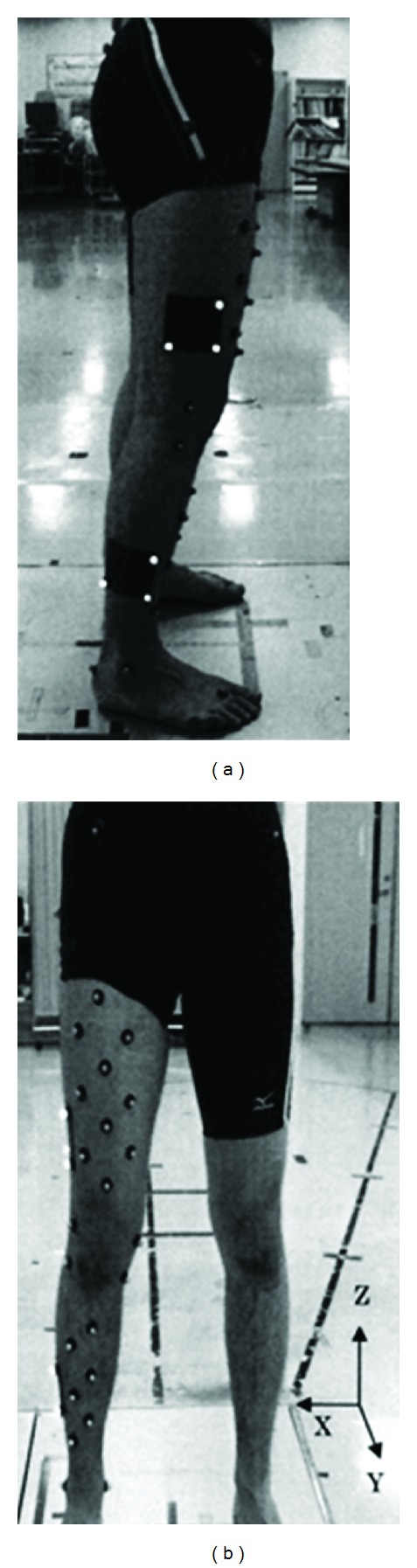
The rigid marker set (black squares with three reflective markers appearing white attached to the thigh and shank) and the point cluster technique (10 markers on the front of the thigh and 6 markers on the front of the shank). Image (a) shows RMS on the lateral side, and image (b) shows the PCT on the front side. The global coordinate system is shown in (b).

A three-dimensional motion analysis system (Vicon, Oxford, UK) and a floor-mounted force plate (Kistler Instruments, Winterthur, Switzerland), each with a sampling rate of 200 Hz, were used in this study. The subjects walked barefoot along a 10 m walkway at their self-selected habitual speeds and were directed to step on a force plate with the right lower limb. Five trials were performed, with sufficient rest between the trials. Reflective markers of 9.5 mm diameter were attached with double-sided tape to each subject's pelvis and anatomical landmarks on the right thigh, shank, and foot segments. After identification by palpation, markers were directly placed over the following anatomical landmarks: bilateral anterior and posterior superior iliac spines, unilateral greater trochanter, lateral and medial femoral epicondyles, lateral and medial tibial condyles, lateral and medial malleoli, calcaneus, and top of the foot at the base of the second metatarsal. Moreover, RMS with three attached reflective markers was placed on the lateral side of the thigh and shank (Figure 1). In addition, 10 markers on the thigh and 6 markers on the shank were attached to calculate the movement of the knee joint by the PCT (Figure 1). After attachment of the markers, decisions were made regarding the relative positions of the anatomical landmarks to the two rigid plates for the RMS and PCT markers based on a single static calibration to estimate the anatomical landmarks of the thigh and shank from the RMS and the PCT markers. The PCT algorithm described by Ida et al. [15] was showed as following. From a rest trial (e.g., quiet standing), a principal axis transformation determines the local reference system that is fixed to the marker cluster. The positions of anatomical landmarks are described on the local reference system by marker cluster. For a dynamic trial, the positions of the anatomical landmarks are extrapolated on the basis of the marker cluster motions during the trial, where the local reference system is calculated for each frame from the marker cluster data. Using the extrapolated anatomical landmark data, the positions of femur and tibia bones are estimated. The estimations of the anatomical landmarks by RMS were calculated with numerical software (Vicon, Bodybuilder) using three markers on each rigid plate.

### 2.3. Data Analysis

Foot strike and toe-off were determined using the force plate data, and the corresponding frame number was identified in the kinematics data. The kinematics data were normalized to the 100% stance phase (foot strike to toe-off = 100%) using spline interpolation. To calculate the kinematics data, the local coordinate systems of the thigh and shank were defined in the three-dimensional position by the anatomical landmarks of the thigh and shank estimated from RMS and the PCT. The knee joint angles during the stance phase were calculated using the joint coordinate system (JCS) approach described by Grood and Suntay [16]. The global coordinate system was defined as follows: the *x*-axis was lateral, *y*-axis was anterior, and *z*-axis was vertical (Figure 1(b)). The coordinate systems for the thigh (*T*) and shank (*S*) were defined as follows:

 
*T*_*x*_: vector directed laterally from the medial to lateral femoral epicondyle,
 
*T*_*y*_: cross product of a vector directed anteriorly from the knee joint center (midpoint between the medial and lateral femoral epicondyles) to the greater trochanter and *T*_*x*_,
 
*T*_*z*_: cross-product of vectors *T*_*x*_ and *T*_*y*_,
 
*S*_*x*_: cross-product of vectors *S*_*y*_ and *S*_*z*_,
 
*S*_*y*_: cross-product of *S*_*z*_ and a vector directed anteriorly from the medial to lateral tibial condyle,
 
*S*_*z*_: vector directed from the ankle joint center (midpoint between the lateral and medial malleoli) to the midpoint between the medial and lateral tibial condyles.

To calculate knee joint angles, two axes of the JCS were embedded in the two segments whose relative motion was to be described. The two vectors were the *T*_*x*_ vector of the thigh coordinate system and the *S*_*z*_ vector of the shank coordinate system. The third axis was called the floating axis and was the common perpendicular to both *T*_*x*_ and *S*_*z*_. Flexion-extension occurred about the *T*_*x*_ axis. The flexion-extension angle, *α*, was obtained by the angle between *T*_*y*_ and the floating axis, and flexion was positive when extension was negative. External-internal rotation occurred about the *S*_*z*_ axis. The external-internal angle, *γ*, was obtained by the angle between *S*_*y*_ and the floating axis, and external was positive when internal was negative. Abduction-adduction occurred about the floating axis. The abduction-adduction angle, *β*, was obtained by the value in which *π*/2 was pulled from *δ* and was defined by the angle between *T*_*x*_ and the *S*_*z*_ axis. Abduction was positive when adduction was negative.

### 2.4. Statistical Analysis

The comparison between RMS and the PCT was examined using Pearson's product moment correlation coefficient for each estimation of the anatomical landmarks in the stance phase. In addition, the coefficient of multiple correlations (CMCs) was used to examine the difference in the angle changes of the knee joint motion in the stance phase provided by RMS and the PCT and was calculated using a method described by Kadaba et al. [17].

## 3. Results

The correlation coefficients for the estimations by RMS and the PCT for the anatomical landmarks in the stance phase are shown in Table 1. The *Y*- and *Z*-coordinates showed very high correlations of ≥0.90 (*P* < 0.01) for all anatomical landmarks. The *X*-coordinates showed slightly lower positive correlations than the *Y*- and *Z*-coordinates. The *X*-coordinates of the medial condyle had a particularly low value of 0.75 (*P* < 0.01).

**Figure 2:**
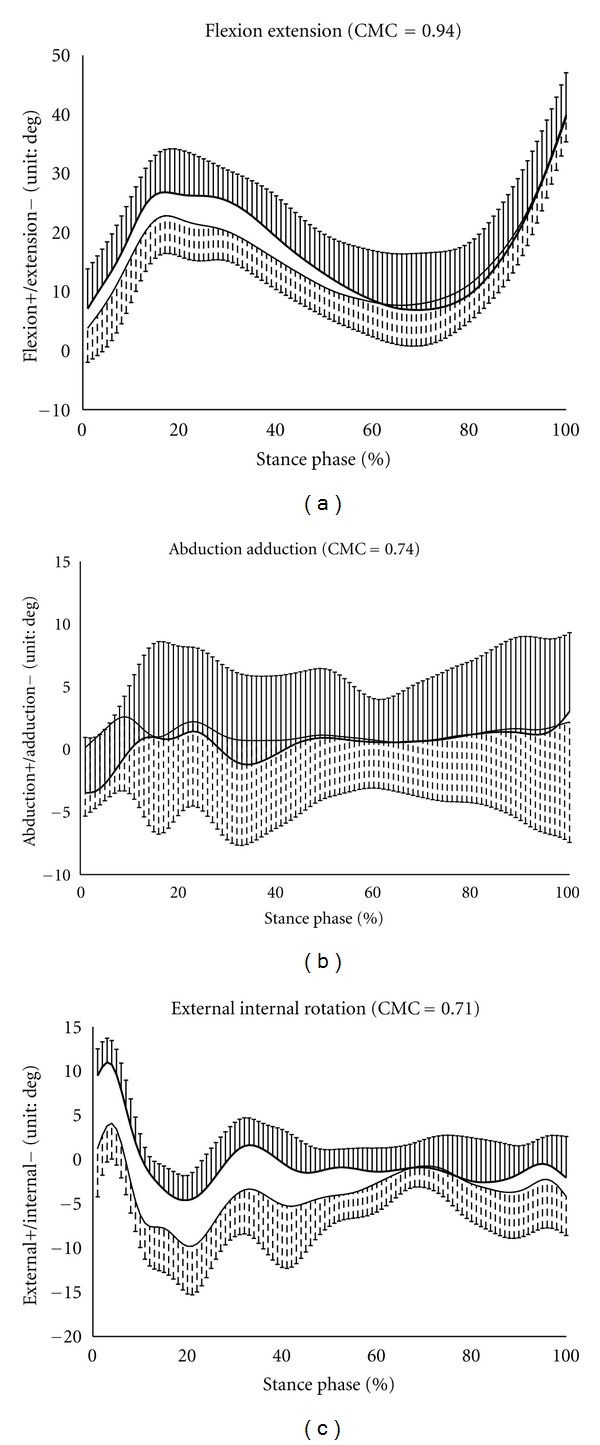
Angle changes of the knee joint motion measured by RMS (thin lines) and the PCT (thick lines). The vertical bars show the SD. Image (a) shows flexion/extension, image (b) shows abduction/adduction, and image (c) shows external/internal rotation. The CMC value is shown in each figure.

The time-dependent changes in the knee joint angles in the stance phase are shown in Figure 2. The data represent the means ± SD for all subjects for the two measurement methods during the stance phase. In these results, the CMC values for the angle changes of the knee joint during the stance phase calculated by RMS and the PCT were 0.94 for flexion/extension, 0.74 for abduction/adduction, and 0.71 for external/internal rotation. As shown in Figure 2, the differences were approximately 4° at 28% of the stance phase in flexion/extension, approximately 1.6° at 5% of the stance phase for abduction/adduction, and approximately 9.8° at the initial contact of the stance phase for external/internal rotation. These results indicate that the differences in the knee joint angles measured by RMS and the PCT were the largest. In terms of external-internal rotation, the error gradually decreased from initial contact to approximately 70% of the stance phase, but after 70%, the error tended to increase again.

## 4. Discussion

This study examined the estimates of anatomical landmarks and the differences in the knee joint motion obtained using RMS and the PCT in the stance phase. The estimates of the anatomical landmarks showed very high positive correlations for the *Y*- and *Z*-coordinates, while the *X*-coordinates showed slightly lower positive correlations than the *Y*- and *Z*-coordinates. The *X*-coordinates of the medial condyle had a particularly low value of 0.75. Because we defined the knee joint center as the link of each middle point of the lateral and medial epicondyles and the long axis of the tibia as the link of each middle point of the lateral and medial malleoli and the middle points of the lateral and medial condyles in this study, the errors in the *X*-coordinates of each anatomical landmark affected the degree of leaning of the long axis of the thigh and shank. This provides the possibility of increasing the error in the angle calculation and is thought to be a factor in why the CMC values for abduction/adduction and external/internal rotation were low compared with the value for flexion/extension.

Based on the data shown in Figure 2, the error grew to 30% from the initial contact in the stance phase between RMS and the PCT. In addition, in terms of rotational motion of the knee joint, the error grew to be large after 70% of the stance phase. The tibialis anterior muscle acts as a brake for plantar flexion of the ankle during the early stance phase. The array of markers used in the PCT is assumed to be affected by the skin deformation caused by contraction of the tibial anterior muscle in the PCT to position the front of the shank. On the other hand, RMS attaches to the outside surface of the shank and is estimated to be affected by contraction of the long peroneus muscle rather than of the tibialis anterior muscle. Because the long peroneus muscle was required for the large contraction during the late stance phase [18], it was suggested that the large contraction of the long peroneus muscle affected the error of rotational motion of the late stance phase.

It was thought that errors might be produced in the anatomical landmark estimates by the *X*-coordinates of the anatomical landmarks estimated from the markers of the shank because the muscles related to the marker attachment position are different. These values may affect the results for abduction/adduction and external/internal rotation. In the early stance phase, the ground reaction force and muscle activity around the thigh become active, and it is thought that large deformation of the skin around the knee is caused by muscle contraction around the thigh. The lateral and medial epicondyles were expected to be particularly affected by muscle contraction around the thigh, and Ishii et al. [19] reported that the medial epicondyle had the largest error compared with the error between the skin marker sets and the PCT. In this study, the lateral and medial epicondyles showed high correlations of >0.90. However, the correlations of the *X*-coordinates were lower than those of the *Y*- and *Z*-coordinates, and this was considered to have an impact on the calculations of the knee angle. In particular, error tended to grow large about the rotational motion of the knee joint during the early and late stance phases; it was supposed that errors of *X*-coordinates of the thigh and shank were strongly affected when the rotational motion of the knee joint grew to be large.

A limitation of this research is that neither RMS nor the PCT follows the motion of a true bone; therefore, true values cannot be calculated. Although the error by skin movement artifacts remains a problem that cannot be avoided in gait analyses using skin-mounted markers, it is suggested that these analyses may obtain comparatively reliable results by using protocols that estimate anatomical landmarks. In the PCT using many reflective markers, it is difficult to attach markers on a few parts because of the influence of muscle contraction. However, for RMS, it is thought that the measurement results can be brought closer to more exact values by setting three markers on parts with as little influence of muscle contraction as possible. In this study, the correlations between protocols of RMS and the PCT were lower for the *X*-coordinates at all anatomical landmarks than for the *Y*- and *Z*-coordinates. In particular, it was difficult to obtain an adequately reliable measurement of the rotational motion of the knee joint, and the errors of *X*-coordinates of each anatomical landmark around the knee joint were a large factor for the rotational motion of the knee joint. However, it is suggested that flexion/extension and abduction/adduction of RMS and the PCT had comparatively little error and good reliability after 30% of the stance phase in this study.

## 5. Conclusions

Flexion/extension and abduction/adduction of the knee by two different protocols had comparatively little error and good reliability after 30% of the stance phase.

## Figures and Tables

**Table 1:** Pearson's product moment correlation coefficients of landmarks estimated by RMS and the PCT.

	*X*	*Y*	*Z*
Great trochanter	0.92*	0.99*	0.95*
Lateral epicondyle	0.86*	0.99*	0.99*
Medial epicondyle	0.85*	0.99*	0.96*
Lateral condyle	0.87*	0.99*	0.99*
Medial condyle	0.75*	0.99*	0.93*
Lateral malleolus	0.87*	0.99*	0.99*
Medial malleolus	0.90*	0.99*	0.99*

^∗^Significant difference was *P* < 0.01 for all landmarks.
